# The Physiological Action of Blood-Letting

**Published:** 1882-08

**Authors:** 


### THE PHYSIOLOGICAL ACTION OF BLOOD-LETTING.

The unquestionable effect of local depletion in relieving some forms of inflammation appears to have been confirmed and explained by the recent researches of Dr. Genzmer, of Halle (*Centr. f. d. Med. Wiss.*, April 1st, 1882). This observer has found that when inflammation has been set up in the web of the frog's foot in the usual way—say by means of a hot wire or by caustics—and the process is watched under the microscope, it is possible to remove the stasis, to empty the blocked vessels, and so far to relieve the inflammation by applying a leech to the limb of the animal between the lesion and the heart. The actual phenomena attending the resolution of the inflammatory process prove, however, to be the very opposite of what might have been expected. Instead of producing anæmia of the affected area, leeching leads to hyperæmia of the part by drawing the blood from the blocked vessels, and allowing a full and rapid stream to flow once more through them. Thus the leucocytes, clinging to the walls previous to diapedesis, are swept away in the blood-current; and one of the elements of inflammation is rapidly removed. But the abstraction of blood causes more than simple resolution. It is manifest that the free influx of blood into the inflamed area—that is, the hyperæmia—must restore the nutrition of the part, the reduction of which constitutes another of the factors of inflammation. Whether or not the leucocytes which may have already escaped from the circulation into the tissues pass back into the vessels, Dr. Genzmer is unable to say. Results similar in kind, but less marked in degree, followed scarification, instead of leeching, between the inflammatory focus and the heart. Distant venesection produced a decidedly less distinct influence. The results of these observations are decidedly valuable, but their importance must not be exaggerated. In the first place, as Dr. Genzmer remarks, they account for the effect of leeching *above* the seat of inflammation, not *at* or *over* it ; secondly, they cannot be said to apply to venesection in visceral inflammations ; and, thirdly, they do not explain the action of leeching or of venesection in the cases where these measures are clinically practised with most success—for example, in cardiac distress or in uræmia. It is possible that the antiphlogistic action of a poultice in inflammation may be the same as the local effect of leeching which has just been described—namely, the reduction of stasis, and the promotion of a free flow of blood through the damaged tissues.—*Medical Times and Gazette,* May 13, 1882.

